# Timing and criteria for prophylactic thyroidectomy in asymptomatic *RET* carriers – the role of Ct serum level

**DOI:** 10.1186/1756-6614-6-S1-S9

**Published:** 2013-03-14

**Authors:** Barbara Jarzab, Sylwia Szpak-Ulczok, Jan Wloch, Agnieszka Czarniecka, Jolanta Krajewska

**Affiliations:** 1 Department of Nuclear Medicine and Endocrine Oncology, Maria Sklodowska-Curie Memorial Cancer Center and Institute of Oncology, Gliwice Branch, Wybrzeze Armii Krajowej 15, 44-101 Gliwice, Poland; 2 Clinic of Oncological and Reconstructive Surgery, Maria Sklodowska-Curie Memorial Cancer Center and Institute of Oncology, Gliwice Branch, Wybrzeze Armii Krajowej 15, 44-101 Gliwice, Poland; 3 present address: Department of Oncological Surgery, University Hospital No.5 of the Medical University of Silesia, Ceglana 35, 40-952 Katowice, Poland

## Abstract

Authors summarize in this brief review results of European discussion, held on ETA-CRN Meeting in Lisbon, 2009, on the American Thyroid Association Medullary Thyroid Cancer (MTC) Guidelines published in the same year and focus on the timing of prophylactic thyroidectomy. ATA 2009 guidelines classified RET protooncogene mutation carriers into 4 levels: A, B, C, D. ATA for prophylactic thyroidectomy were generally independent of the serum calcitonin (Ct) concentration but based on *a priori* risk levels. This was well accepted as the important novelty was to delineate risk level specially for RET 634 mutation (level C). In the ATA Guidelines total prophylactic thyroidectomy below age 5 years was recommended in RET 634 mutation carriers regardless of Ct status. However, some European experts favored to base the decision not only on the results of DNA testing but also on the going Ct level. The European discussion reflected divergent opinions and indicated the need of publication of European experience instead of arbitrary opinions. It was stressed that patients carrying the same RET mutation present heterogenic progression to the clinically overt medullary thyroid cancer, even in the same family. Thus, in summary, the ATA MTC guidelines constituted a positive stimulus to publish further evidence for Ct-guided pre-emptive thyroidectomy for RET gene mutation carriers and the conclusion is drawn on the basis of experience expressed in Lisbon and published later evidence that the integrated algorithm based on age - Ct - type of RET mutation should be considered in the decision of pre-emptive thyroidectomy.

## 

In 1993 germline mutations of *RET* were discovered in hereditary medullary thyroid cancer [1,2]. Within a few years DNA testing of protooncogene *RET* mutations was proposed to patients and their families [3]. To avoid medullary thyroid cancer development prophylactic (pre-emptive) thyroidectomy was indicated in identified mutated *RET* gene carriers [4]. A few years later, in 2001 for diagnosis and therapy of MEN [5], known as Gubbio consensus, proposed to relate the timing of prophylactic thyroid operation to the type of mutation depending on three defined levels of risk: 1, 2 and 3. In summary, in MEN 2B patients (level 3, *RET* 918) prophylactic thyroidectomy with central lymph node dissection (extended lymphadenectomy in the case of lymph node metastasis identification) was recommended within first 6 months of life (preferably within the first month). Young patients with *RET* 611, 618, 620 and 634 mutations were classified as level 2 of MTC risk in whom prophylactic thyroid operation should be performed before the age 5 years. The extension of lymphadenectomy for each level of risk was not equivocally indicated. Level 1 included 609, 768, 790, 791, 804, and 891 *RET* mutations with the lowest risk of MTC. Different strategies for level 1 were presented: 1) prophylactic thyroidectomy before age 5, 2) prophylactic thyroidectomy before the age 10, 3) prophylactic thyroidectomy at the time of abnormal result of pentagastrin-stimulated Ct test.

In 2009 American Thyroid Association published guidelines [6] where the risk of medullary thyroid cancer was classified into 4 levels: A, B, C, D. ATA for prophylactic thyroidectomy were generally independent of serum calcitonin (Ct) concentration but based on *a priori* risk levels. Level D includes MEN 2B cases where prophylactic thyroidectomy is recommended within the first year of life, as soon as possible. In children above one year of age prophylactic dissection of the central lymph node compartment is recommended. The ATA guidelines indicated that there was insufficient data to recommend central lymphadenectomy in MEN 2B children below 1 year and warned that the normal range of serum Ct concentration for small children was unknown.

The important novelty was to delineate risk level specially for *RET* 634 mutation (level C). Total thyroidectomy below 5 years was recommended in *RET* 634 mutation carriers regardless of Ct status. In level B (*RET* 611, 618, 620) thyroid surgery should be done before the age 5 years. In level B and A (rare *RET* mutations with low gene penetrance ex. *RET* 768, 790, 804, 891) thyroidectomy could be postponed beyond the age 5 years in families with less aggressive MTC history and in consideration of family preference, if basal ± stimulated serum Ct and thyroid ultrasound were normal. ATA recommended central and lateral lymphadenectomy in levels A-C in the case of clinically evident lymph node metastases (image or biopsy positive).

This subdivision to four risk levels was generally very well accepted, because all felt the need to differ *RET* 634 mutation from other exon 10/11 mutations [7]. However, the indications to prophylactic thyroidectomy were evaluated as arbitrary. Already after publication of the ATA Guidelines, in 2012 Elisei and co-workers [8] presented a study of 84 *RET* gene mutation carriers with MEN 2A and FMTC where thyroidectomy was proposed to *RET* mutation carriers with elevated serum Ct concentrations independently of the age and type of *RET* mutation status. Patients with basal and/or stimulated elevated Ct (both > 10 pg/ml, equal to >10 ng/L) were referred to thyroid operation. Among the presented group there were 21 patients with elevated basal Ct at the moment of their *RET* mutation status identification. Thirty one *RET* gene carriers presented normal basal Ct levels with a positive pentagastrin test and thirty two patients were basal Ct negative during follow-up (for 2-16 years). The authors did not observe any correlation between the age of gene carriers’ or with type of *RET* mutation and basal Ct level. Similarly, no correlation between serum Ct and the ATA risk level (A, B or C) was observed at the time of thyroidectomy. The authors decided on the basis of elevated Ct level and fifty one patients with elevated basal and/or stimulated Ct level underwent thyroidectomy. All patients with basal hypercalcitoninaemia were diagnosed with MTC. In the group with elevated stimulated Ct 25 of 31 patients were diagnosed with MTC but all in stadium pT1N0 (tumor diameter 0.38 cm ± 0,1) and the rest (6/31) were diagnosed with C cell hyperplasia. The authors also analyzed the risk of lymph node metastases in correlation to the basal Ct level. The proposed cut-off for basal Ct of less than 60 pg/ml (60 ng/L) unequivocally distinguished patients without lymph node involvement (sensitivity 100%, specificity 86,5%). It may be concluded, that authors preferred decisions based on serum Ct than proposed ATA risk levels.

Machens and Dralle in their editorial published in Thyroid in 2009 [9] point out that patients carrying the same mutation even in the same family present heterogenic progression to clinically overt medullary thyroid cancer. Authors propose the integrated DNA and biochemical based concept of prophylactic thyroidectomy. The moment of C-cell transition to medullary thyroid cancer usually occurs when stimulated Ct level rises. When basal Ct starts to increase, the opportunity of medullary thyroid cancer significantly occurs. Authors present skeptic evaluation of ATA absolute thresholds for systemic lymph node dissection. The recommended cut-offs: primary tumor size of 5 mm and basal Ct of 40 pg/ml need further evaluation and confirmation in the independent studies.

Also, Rohmer et al. [10] performed with the French Group of Endocrine Tumors a retrospective multicentre analysis of 170 young (under 21) patients from hereditary MTC families. Retrospective analysis included patients operated from 1977 to 2006. The *RET* status in many cases was assessed retrospectively. The age at thyroidectomy and its extension were dependent on the type of mutation (if available) and preoperative Ct serum level. In this group 92.4% and 38.3% of patients had elevated stimulated and basal Ct, respectively. All 170 patients underwent total thyroidectomy, among them 106 with central and 33 with additionally lateral lymph node dissection. Regardless of the ATA risk level all patients operated with basal Ct below 30 pg/ml (30 ng/L) were cured. The authors suggested that time of prophylactic thyroidectomy should always be dependent on preoperative Ct serum concentration rather than risk class with exception of MEN 2B where operation should be performed as soon as possible. Similarly a Ct serum concentration above 10 pg/ml was proposed cut-off for lymph node dissection. Rohmer and co-workers did not find any improvement when analyzing stimulated Ct compared to its basal concentration.

Similar position is presented in a retrospective study by Wloch, 2007 [11] where 119 *RET* carriers underwent thyroidectomy up to 2007. The decision about the age and extent of prophylactic thyroidectomy in *RET* gene carriers was individualized depending on Ct level. The increase of Ct level was a major factor determining the indication to earlier surgery. In case of familial history suggesting a mild phenotype of disease (especially patients with mutation in codon 791) author postponed the decision about surgery up to age of 5-10 years, and in some families even to the age of 10-18 assumed that stimulated Ct was rigorously monitored.

In fact, in many studies Ct proved to be a good cancer marker. Its concentration was strictly correlated with tumor mass [12]. When basal and stimulated Ct levels were within normal range histopathological examination usually revealed at most C-cell hyperplasia. In the patients showing positive stimulation test C-cell hyperplasia or medullary microcarcinoma was diagnosed. Increasing Ct level (both basal and stimulated) was correlated with more advanced medullary cancer. Also, the risk of central nodes metastases, lateral nodes involvement and distant metastases was correlated with high Ct level [10]. This problem has been discussed within the ETA-CRN Meeting in Lisbon 2009 [13]. Based on a valuable paper published by Machens et al. in NEJM in 2003 [14] it is important that all European experts were like-minded to support ATA risk classes and considered it to be beneficial to *RET* mutation carriers, but the decision about the timing of prophylactic thyroidectomy needed to be reinforced by the Ct serum level. Irrespective of the availability of pentagastrin the Ct stimulation test in this setting continues to play an important role [13]. In summary, considering recently published evidence [8,10] for the timing of pre-emptive thyroidectomy it may be concluded, that the ATA MTC guidelines constituted a positive stimulus to publish further evidence for Ct-guided preemptive thyroidectomy for *RET* gene mutation carriers and the conclusion is drawn on the basis of experience expressed in Lisbon and published later evidence that the integrated algorithm based on age - calcitonin - type of *RET* mutation should be considered at the decision of pre-emptive thyroidectomy.

## List of abbreviations used

Ct: Calcitonin; MTC: medullary thyroid carcinoma.

## Competing interests

Authors declare no conflicts of interests.

## Declarations

This article has been published as part of *Thyroid Research* Volume 6 Supplement 1, 2013: European comments on Medullary Thyroid Cancer Management Guidelines of the American Thyroid Association. The full contents of the supplement are available online at http://www.thyroidresearchjournal.com/supplements/6/S1. Publication of this supplement has been supported by the European Thyroid Association-Cancer Research Network.
